# Functionally Brain Network Connected to the Retrosplenial Cortex of Rats Revealed by 7T fMRI

**DOI:** 10.1371/journal.pone.0146535

**Published:** 2016-01-08

**Authors:** Jingjuan Wang, Binbin Nie, Shaofeng Duan, Haitao Zhu, Hua Liu, Baoci Shan

**Affiliations:** 1 Division of Nuclear Technology and Applications, Institute of High Energy Physics, Chinese Academy of Sciences, Beijing, China; 2 Beijing Engineering Research Center of Radiographic Techniques and Equipment, Beijing, China; Shenzhen institutes of advanced technology, CHINA

## Abstract

Functional networks are regarded as important mechanisms for increasing our understanding of brain function in healthy and diseased states, and increased interest has been focused on extending the study of functional networks to animal models because such models provide a functional understanding of disease progression, therapy and repair. In rodents, the retrosplenial cortex (RSC) is an important cortical region because it has a large size and presents transitional patterns of lamination between the neocortex and archicortex. In addition, a number of invasive studies have highlighted the importance of the RSC for many functions. However, the network based on the RSC in rodents remains unclear. Based on the critical importance of the RSC, we defined the bilateral RSCs as two regions of interest and estimated the network based on the RSC. The results showed that the related regions include the parietal association cortex, hippocampus, thalamus nucleus, midbrain structures, and hypothalamic mammillary bodies. Our findings indicate two possible major networks: a sensory-cognitive network that has a hub in the RSCs and processes sensory information, spatial learning, and episodic memory; and a second network that is involved in the regulation of visceral functions and arousal. In addition, functional asymmetry between the bilateral RSCs was observed.

## Introduction

The human brain is a complex hierarchical network capable of highly functional integration and segregation [1]. The widely separated regions of the human brain exhibit a distinct functional network connected by interregional associations. Distinct networks, such as the vision network, motor network, auditory network, language network, and default-mode network [2–4], have been consistently identified in humans. In addition, disruptions to these functional networks are suggested to cause pathological states [5]. Thus, functional networks can be used as an important noninvasive tool for furthering our understanding of brain functions in healthy and diseased states.

There is increased interest in extending the study of functional networks to animal models because such models can provide a functional understanding of disease progression, therapy and repair. In rodents, the retrosplenial cortex (RSC) is an important cortical region that extends over half the length of the entire cerebrum; thus, it is one of the largest cortical regions in rodents. Neuroanatomically, the RSC is regarded as an ‘intermediate’ cortex because it has a transitional pattern of lamination between the neocortex and archicortex [6]. A number of invasive studies have highlighted the vital role of the RSC in many functions, including spatial memory [7,8], processing multiple stimuli simultaneously [9], episodic memory [10], imagination and thinking about the future [6]. However, the functional network based on the RSC in rodents remains unclear. Because of the critical importance of the RSC, we speculate that there may be a network based on the RSC that plays an important role in executing brain functions.

In the present study, we defined the bilateral RSCs as two regions of interest and investigated whether there is a network based on the RSC.

## Experimental Procedures

### Subjects

Thirty healthy adult Sprague Dawley (SD) rats (9 females; age range 9–11 weeks old; weight range 250–300 g) were obtained from Shanghai Lab Animal Research Center. All experiments were performed with the approval of the Animal Care and Use Committee of the Chinese Academy of Sciences and conformed to named international guidelines on the ethical use of animals. All animals had ad libitum access to food and water throughout the experiment and were subjected to a controlled 12-h light: dark schedule (lights on at 07:00). Prior to MRI scanning, the rats were initially anesthetized using 3–4% isoflurane in a 1:4 oxygen and air mixture. During the MRI scan, the animals were placed prone in a MR-compatible stereotactic holder with the head cinched, the teeth placed firmly in a tooth bar, and the nose emplaced in a nose cone to exhaust isoflurane in a mixture of oxygen and air (ratio 1:4). After insertion into the MRI scanner, the animals’ physiological conditions, including their body temperature, pulse (using a pulse oximeter) and respiration rate, were monitored (SA Instruments, Stony Brook, NY, USA). The core body temperature was controlled to 37°C using a feedback-controlled warm air system (SA Instruments, Stony Brook, NY, USA). Respiration was maintained at a rate of 50 breaths per min.

The animals were used only once and sacrificed at the end of the experiments using a urethane anesthetic at a dose three times greater than that of the isoflurane used during the scan.

### Data acquisition

Functional images were acquired on a 7.0 T animal MRI scanner (70/16 PharmaScan, Bruker Biospin GmbH, Germany) in Nanjing using a 38-mm birdcage rat brain quadrature resonator for radiofrequency transmission and receipt. The images were obtained with an echo planar imaging (EPI) sequence (TR = 2 s, TE = 18.73 ms, matrix size = 96 * 96 * 27, voxel size = 0.26 * 0.21 * 1 mm^3^, flip angle = 90^0^, slice gap = 0, and total volumes = 120). All the original Bruker images were converted to DICOM format with the software program (Paravision 5.1) included with the scanner.

### Data analysis

To identify the whole brain connectivity of the retrosplenial cortex, we defined the bilateral retrosplenial cortices as regions of interest based on the rat atlas [11]. Unless specifically stated otherwise, all the preprocessing was conducted using our in-house software spmratIHEP [11] for voxel-wise analyses of the rat brain images. (1) Slice timing: the images were first corrected for the acquisition time delay among different slices; (2) Realignment: all the individual functional images were realigned to the first volume to correct for head movement; (3) Normalization: the voxel size of the individual images was magnified five times to match the size of the human brain; this magnification did not generate any changes in the matrix. The images were then standardized to the Paxinos & Watson space [12]. (4) Smoothing: the spatially normalized functional images were smoothed by a 2 * 4 * 2 Gaussian kernel with full width at half-maximum (FWHM).

Using DPARSF (http://rfmri.org/DPARSF), all smoothed images were then 0.01–0.1 Hz band-pass filtered and further corrected for the effect of head movement by regressing the translations and rotations of the head estimated during image realignment. We evaluated the functional connectivity using seed-based correlational analyses on a voxel-by-voxel basis [13,14]. The time courses from all voxels within the individual seed regions were averaged and used as reference time courses. Pearson’s cross-correlation coefficients between these reference time courses and the time course of each individual voxel were then calculated and used to quantify the strength of the functional connectivity. The correlation coefficients then underwent Fisher’s z-score transformation within the mask. For each rat, a functional connectivity map was established.

### Statistics analysis

We chose 0.2027 as the Z threshold value, which corresponded to a correlation coefficient of 0.2 [13]. If the Z-score exceeded the Z threshold value, the voxel was present; otherwise, it was not present. At the group level, a voxel-wise one-sample t-test was performed to estimate the seed-based connectivity. Functional connectivity with significance was determined based on a voxel-level height threshold of p < 0.001 (FWE corrected) and a cluster-extent threshold of 50 contiguous voxels. We extracted the related significant regions from the threshold results via the rat atlas and then quantitatively evaluated the number of voxels and the mean connection coefficient values in every significant region. At the region level, we calculated the average T value.

## Results

**Fig 1:**
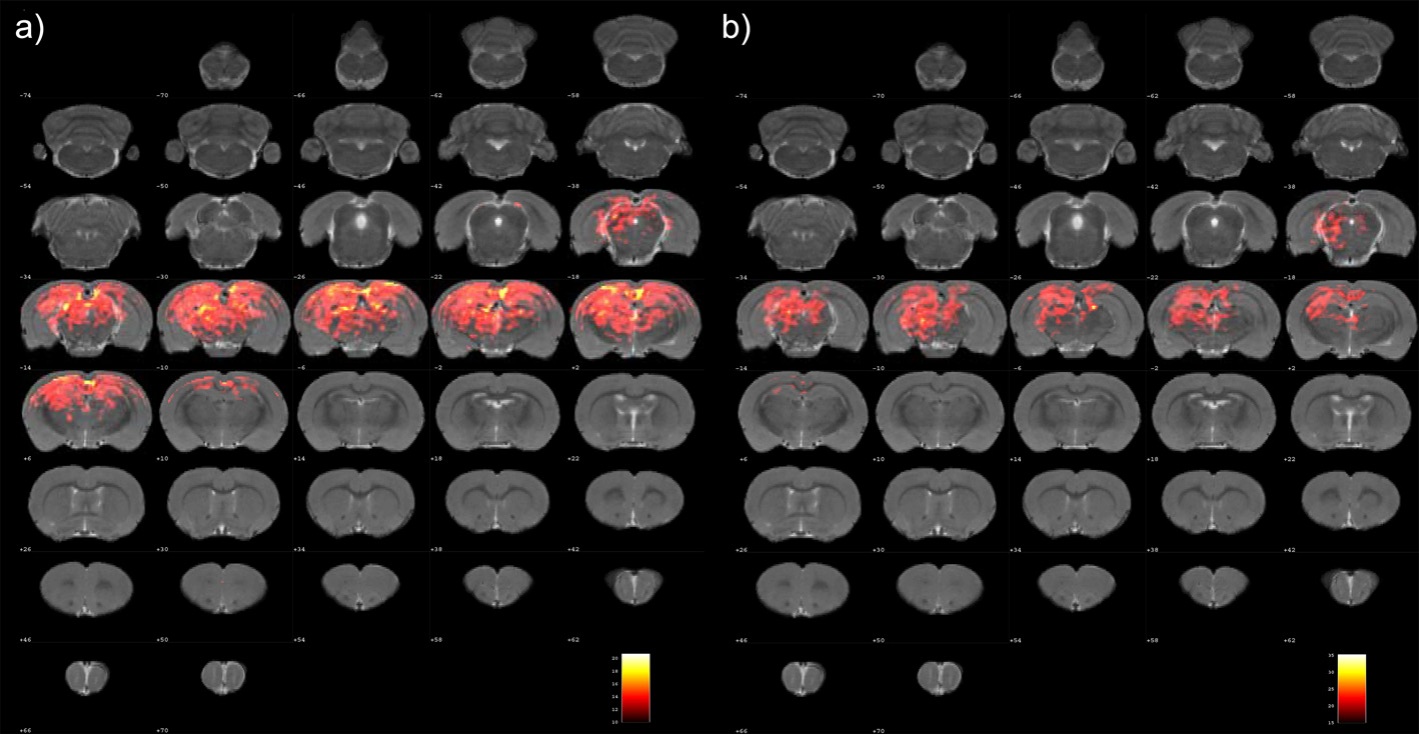
Brain regions with significant connections with the bilateral RSCs in the anesthetized rats: (a) left RSC and (b) right RSC. These significant regions were shown in coronal slices as a color-coded statistical T-values superimposed on a set of normalized coronal atlas of the rat brain. RSC, the retrosplenial cortex.

**Fig 2:**
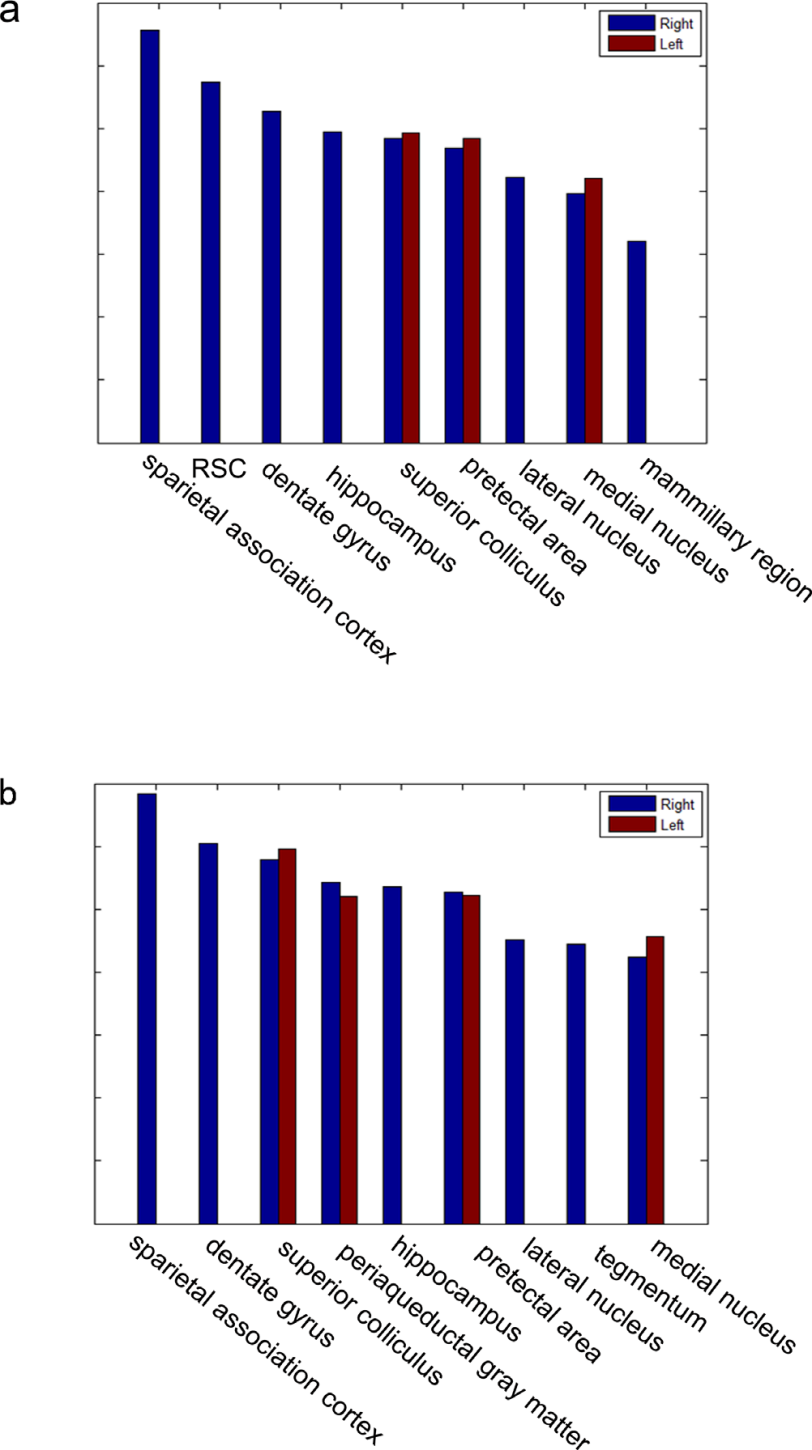
Z value of the functional connectivity between the bilateral RSCs: (a) left RSC and (b) right RSC and other regions. The blue color represents the regions located in the right brain, and the red color represents the regions located in the left brain.

**Fig 3:**
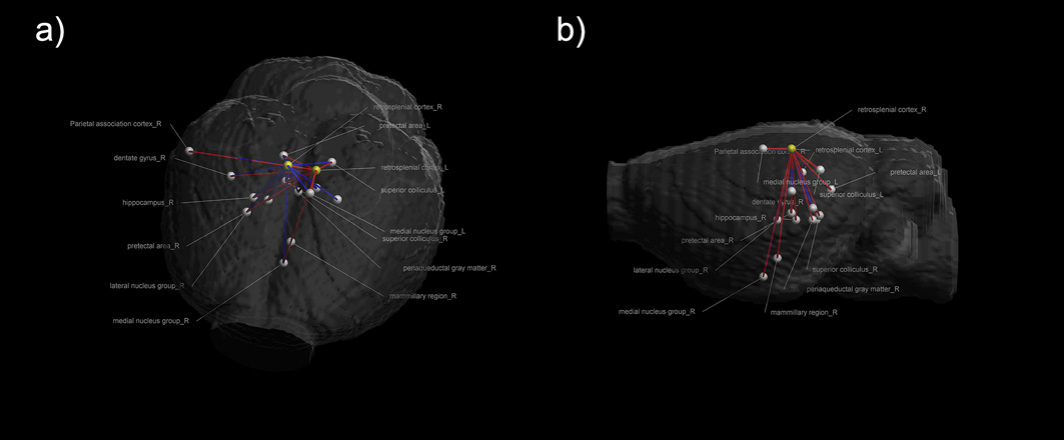
Views of functional networks with the bilateral RSCs shown in the three-dimensional rat brain model from two directions: (a) from top to down and (b) from right to left. The two nodes presented in yellow represent the bilateral RSCs. The white color indicates all of the related regions which that are functionally connected to the bilateral RSCs. The red and blue lines represent the functional connections with the left and right RSCs, respectively.

Significant seed-based connectivity results were investigated in the anesthetized rat. On both sides of the RSC, widespread connections were observed between the cortex and the sub-cortex. We displayed the T maps on a representative rat brain model to facilitate the visualization of spatial relationships (Fig 1), and we also provided the average T value in the significant regions instead of the current maximum peak value. The regions with high T values mainly included the parietal association cortex, hippocampus, thalamus nucleus, midbrain structures, and hypothalamic mammillary bodies. In addition, Paxinos and Watson’s coordinates were used to provide detailed visualizations of the brain regions that presented significant connectivity with the bilateral RSCs (Tables 1 and 2). Fig 2 shows that the related regions were functionally connected with the a) left RSC and b) right RSC and arranged by connectional strength in descending order. To facilitate visual comparisons, we included the significant connections with the bilateral RSCs in the three-dimensional brain model (Fig 3).

**Table 1:** Brain regions with significant functional connectivity with the left RSC.

ROI	Mean T	Num	X	Y	Z
			Paxinos Coordinates		
Midbrain_pretectal area right	22.335	28	-2.219	4.443	-4.677
Hypothalamus_mammillary_region right	20.155	15	-0.491	7.254	-3.957
Midbrain_superior colliculus right	19.454	1607	-1.938	4.544	-5.637
Parietal association cortex right	19.387	232	-2.636	0.663	-3.477
Midbrain_pretectal area left	19.146	22	1.927	4.996	-4.917
Dorsal thalamus_medial nucleus right	18.967	509	-0.364	5.467	-3.447
Dorsal thalamus_lateral nucleus right	18.795	4953	-3.675	6.342	-5.637
Dentate gyrus right	18.008	57	-4.330	4.782	-6.597
Dorsal thalamus_medial nucleus left	17.777	497	-0.097	5.628	-3.477
Midbrain_superior colliculus left	17.353	1606	0.476	3.140	-6.357
Hippocampus right	17.226	5104	-2.466	2.949	-6.117
Retrosplenial cortex right	16.836	3213	-0.107	1.038	-2.758

**Table 2:** Brain regions with significant functional connectivity with the right RSC.

ROI	Mean T	Num	X	Y	Z
			Paxinos Coordinates		
Midbrain_pretectal area right	29.961	28	-2.212	4.778	-5.16
Midbrain_superior colliculus right	26.0059	1607	-2.072	4.683	-5.638
Dentate gyrus right	25.253	57	-4.201	4.623	-6.358
Midbrain_superior colliculus left	23.238	1606	1.276	4.918	-6.118
Parietal association cortex right	21.899	232	-3.167	0.654	-3.718
Dorsal thalamus_lateral nucleus right	21.613	4992	-3.676	6.342	-5.638
Midbrain_pretectal area left	21.476	22	2.064	5.025	-5.158
Tegmentum of midbrain right	21.22	3054	-2.072	6.727	-5.638
Hippocampus right	20.914	5146	-1.023	2.864	-4.198
Dorsal thalamus medial nucleus right	20.648	509	-1.430	4.989	-3.718
Midbrain periaqueductal gray matter left	20.585	576	0.467	5.705	-5.638
Dorsal thalamus medial nucleus left	20.538	4997	0.307	5.67	-3.718
Midbrain periaqueductal gray matter right	20.058	572	-0.736	5.198	-5.638

## Discussion

In this study, we defined the RSCs as two regions of interest and investigated their functional connectivity with other cerebral voxels. The results showed that the related regions mainly included the parietal association cortex, hippocampus, thalamus nucleus, midbrain structures, and hypothalamic mammillary bodies. Moreover, our results were consistent with the known anatomical connections of RSCs in rats [6].

The RSC has an important role in many cognitive functions, including spatial learning and episodic memory [6]. Brain function depends on functional networks formed by the association of related regions. Acting as a main node, the thalamus receives relatively raw sensory information and projects this information to a wide range of cortical areas, such as the frontal lobe and parietal cortex, for further processing. Raw sensory information can be further integrated in the parietal cortex and other cortical areas. After integration, sensation from the thalamus might be converted to perception as a component of the learning process. Learned inputs may then be sent to the storage system by the hippocampus-centered memory network for instant working and later retrieval tasks. In short, such a simplified and vague framework of the general sensory-cognitive pathway is outlined based on the general functions of those three neural modules without considering modulation by the RSC. According to the results of this study, the RSC has close functional connections with all three areas. RSC lesions in rodents impair spatial memory tasks, including learning fixed locations [15] or daily changed locations of a platform in a water maze and performing working memory tasks in a radial-arm maze [8]. We propose that the RSC is involved in all the main stages of information processing, from sensation to learning and memory, and we further hypothesize that the RSC is a main hub and not just a node in the sensory-cognitive network. Such a view is also supported by other studies on functional connectivity and the default mode system [16,17]. Although we cannot speculate on the specific interaction between RSCs and each region, RSCs might play an important role in modulating the sensory-cognitive network as a hub. In addition, studies have reported that the organization of brain networks varies across cognitive states and time [18,19]. In the present study, the animals were in a state of anesthesia without explicit tasks; thus, we hypothesized that the sensory-cognitive network based on RSCs is intrinsic. The sensory-cognitive network centered on the RSC in this study remains relatively stable, although various networks subtly rearrange and reconfigure themselves dynamically to support special cognitive functions [20]. Overall, we hypothesized that there is a sensory-cognitive network that processes sensory information, spatial learning, and episodic memory and that it includes the parietal cortex, RSC, thalamus nucleus, and hippocampus and has a hub at the RSC.

In addition, our study indicated that the anesthetized rat lost cognitive function but maintained stable basic vital signs. The RSC has been defined as the ‘visceral brain,’ and its stimulation can evoke autonomic changes [21]. Our results showed that the RSC had significant functional connectivity with the related regions of the midbrain reticular formation. The midbrain reticular formation, which is distributed in the tegmentum and periaqueductal gray, is responsible for regulating visceral sensations [22]. Physiological experiments have found that disturbances to the midbrain reticular formation impair breathing and promote changes to the heart rate, and these alterations may be life threatening [23]. Thus, the functional connectivity between the RSC and midbrain reticular formation may confirm cooperation among related regions. In addition, we found that the RSC had also functional connectivity with the dorsal thalamus lateral nucleus, dorsal thalamus medial nucleus and mammillary bodies. Acetylcholine released by the tegmentum of the midbrain activates the reticular thalamic nucleus, which plays an important role in arousal [24]. In our applied atlas, the reticular thalamic nucleus is included in the dorsal thalamus lateral nucleus. The functional connectivity between the RSC and dorsal thalamus lateral nucleus may be facilitated by the reticular thalamic nucleus. Disruption to the mammillary bodies causes a failure to switch from sleep to wakefulness [25]. Thus, from the functional evidence mentioned above, we speculated that the network of the midbrain structures, RSC, thalamus nucleus, and mammillary bodies is involved in the regulation of the autonomic nervous system.

In our study, we defined two regions of interest: the right RSC and left RSC. As shown in Fig 2, we found that the bilateral RSCs had widespread functional connections with regions in the right hemisphere, and regions in the left hemisphere all had corresponding regions in the right hemisphere. Previous studies have reported the structural asymmetry between the hemispheres of the rat brain [26]. Structural asymmetry, which is also found in the human brain, may be the neuroanatomical basis of the functional asymmetry observed in our results. The hemisphere asymmetries detected in the human brain are hypothesized to be the result of evolution. In contrast, temporal lobe asymmetries may also be the key to the etiology of schizophrenia [27]. In addition, extensive connections across brain hemispheres were also found, and we speculated that these connections may reinforce the cooperative transmission of information across brain hemispheres.

The present study has several methodological limitations. Our study was performed under anesthesia, which has been reported to change the strength of the functional connections between brain regions [28]. However, under the effect of isoflurane, we also found significant functional connections. Future studies are needed to confirm whether these results can be generalized to other anesthetic agents and/or different dosages.

## Conclusions

In summary, we found that the functional networks based on the RSC mainly include the parietal association cortex, hippocampus, thalamus nucleus, midbrain structures, and hypothalamic mammillary bodies. Our findings suggest that there were two major networks, and functional asymmetry between the bilateral RSCs was observed.
